# Descriptive Analysis of Resources Used to Learn About Residency Programs Since Transition to Virtual Interviews

**DOI:** 10.5811/westjem.33574

**Published:** 2025-05-19

**Authors:** Richard Bounds, John Priester, Benjamin Lewis, Roz King, Skyler Lentz

**Affiliations:** University of Vermont Health Network, Department of Emergency Medicine, Burlington, Vermont

## Abstract

**Introduction:**

The transition to virtual interviews over the past four years has been associated with changes to the ways that applicants collect information on residency programs.

**Methods:**

Our program collected free-response data from questionnaires completed by applicants prior to their virtual interview days over the course of four recruitment cycles. We performed a descriptive analysis of these responses to identify the frequency with which students have been accessing various resources to learn about programs, and to learn how that has changed over time.

**Results:**

Our findings over four years and 322 applicants (of 391 surveyed, response rate 82%) indicated that the three most common sources of information were individual program websites, the Emergency Medicine Resident’s Association (EMRA) Match website, and Instagram. These sources were reported more frequently than personal experience, word of mouth, and advice from mentors. Other online resources were rarely used.

**Conclusion:**

These findings may help program leaders to direct their limited time and attention towards marketing their programs through online resources most commonly used by applicants.

## INTRODUCTION

Since the onset of the pandemic in 2020, residency application and interview processes have changed dramatically. The transition from in-person to virtual interviews and the decreased availability of visiting rotations has created challenges for students in assessing programs and experiencing their unique elements.^1^–^3^ With the rise in use of social media and various online platforms for sharing information and interacting with programs, medical students may place greater value on these web-based resources than advisor recommendations, word-of-mouth, and official websites endorsed by national organizations. An awareness of applicants’ level of engagement with various resources exploring residency programs could help program leaders direct their time and attention toward those resources most commonly used by applicants. We aimed to provide a descriptive analysis of the resources used by applicants to our program, and how their use has evolved over the last four years, to better inform program leaders in their ongoing recruitment efforts.

## METHODS

We conducted this study at our three-year, academic program in the Northeast. The emergency medicine (EM) residency program began in 2019 and accepts six residents each year. Over the course of four consecutive academic years (2020–2024), our program coordinator sent a brief electronic questionnaire to each applicant prior to their interview day. In addition to asking (1) why applicants were interested in our program and (2) whether they were interested in any specialty areas of emergency medicine, our survey also asked (3) “What resources have you used to learn more about our program?” Responses to the first two questions were provided to interviewers to make our short virtual interviews more efficient. Free-text responses to the third question were reviewed in aggregate by our selection committee at the end of each interview season in an effort to best inform our future program recruitment efforts. After recognizing trends over the past four years, we decided to perform a descriptive analysis to share our findings.

All responses were deidentified, and this project was found to be exempt from institutional review board review. To measure the frequency of reported resources used from our free-text responses, we used the Linguistic Inquiry and Word Count (LIWC) (https://www.liwc.app) program. This validated text analysis software program analyzes text files and compares words within a text document to predefined word dictionaries, customized by the study team. For example, the category “Conference” included the words: fair, meeting, conference, American College of Emergency physicians (ACEP), and Society of Academic Emergency Medicine (SAEM). In creating the dictionary for each category of resources, two investigators reviewed the responses to reach agreement. (See Appendix for customized dictionary)

## RESULTS

**Figure 1:**
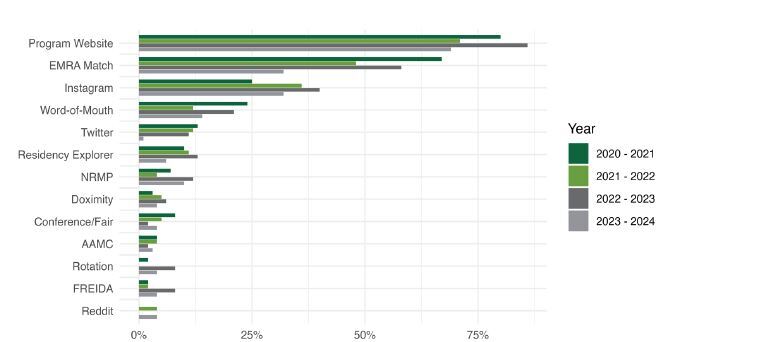
Proportion of applicants that reported using various resources to learn about residency program. *EMRA*, Emergency Medicine Residents’ Association, *NRMP*, National Resident Matching Program; *FREIDA*, Fellowship and Residency Electronic Interactive Datase; *AAMC*, Association of American Medical Colleges.

Over four years, our pre-interview survey was sent to 391 medical student applicants selected to interview at our program and we collected responses from 322 (response rate 82%). Figure 1 shows the proportion of applicants that reported using various resources for learning about our program.

The residency program’s website continues to be the most frequently used resource. The Emergency Medicine Residents Association (EMRA) Match website has been a close second but seems to be decreasing in the proportion of applicants that use this site. Instagram use appears to be rising slightly each year. All three of these resources (program website, EMRA Match website, and Instagram) were reported to be used more frequently than personal experience, word of mouth, conversations with peers, and advising from mentors. Other sites including Residency Explorer, National Resident Matching ProgramRMP, Doximity, Association of American Medical CollegesAAMC, Fellowship and Residency Electronic Interactive Datase (FREIDA), Twitter, and Reddit were used by a small minority of applicants.

## DISCUSSION

Since the pandemic, the shift to virtual interviews has been viewed favorably by medical students, with advantages including decreased costs, fewer scheduling conflicts, decreased carbon footprint, and the opportunity to easily interview with programs across geographically distant locations.^4^ However, various challenges have arisen, particularly regarding residency programs’ ability to attract highly qualified candidates in the absence of in-person experiences. Residency applicants have expressed that the inability to personally experience programs and their cities is a significant drawback in virtual interviews.^3^ One strategy to address these challenges has been an increased reliance on web-based resources such as program websites and social media platforms as a way for residency programs to market their programs and interact with potential applicants.^5^ Our findings indicate that these online platforms have supplanted other resources including medical school advisors, word-of-mouth recommendations, and informational websites such as the American Medical Association FREIDA database. As our program grew from newly established in 2019, to two years of graduates in 2023, these findings did not appreciably change.

**Population Health Research Capsule**

What do we already know about this issue?

*The transition to virtual interviews over the past four years has been associated with changes in ways that applicants collect information on residency programs.*

What was the research question?

*We identified the frequency with which students access information about programs from various resources.*

What was the major finding of the study?

*With a response rate of 82%, we found that the three most common resources were individual program websites, the Emergency Medicine Resident’s Association (EMRA) Match website, and Instagram.*

How does this improve population health?

*These findings might help program leaders to direct their efforts towards those resources that might have the greatest impact on recruitment.*

Prior work has described the factors influencing residency selection, although fewer studies have described resources that students use when determining which programs to apply to.^6^,^7^ Medical students have relied on residency program websites for over two decades, making them one of the longest-standing available resources. Various studies from 2002–2005 surveying applicants to emergency medicine and anesthesia reported that the program website was an important factor in their decisions to both apply to and rank programs.^8^–^10^

The rise of social media use by medical students over the past decade has created an additional tool for the dissemination of information by residency programs when recruiting potential applicants.^11^,^12^ Medical students are increasingly using social media, in addition to program websites and other resources, to gather information from residency programs. In a 2020 study of medical students applying to anesthesia programs, the most used resources when researching residency programs included program websites (91.5% of applicants) as well as social media platforms including Doximity (47.6%), Instagram (38.5%), and X (formerly Twitter, 19.4%).^13^ Similarly, for medical students applying to general surgery residency programs in the 2020–2021 application cycle, residency program websites were the most used resource (92.4% of applicants), followed by social media platforms including Doximity (36.5%), X (35.6%), Instagram (33.7%) and Facebook (11.6%).^14^ The relatively low use of X may be explained by our program’s discontinuation of active use in 2022.

Additional shifts in social media use have been documented during and following the COVID-19 pandemic. For example, the use of X increased from 21% to 41% in matched applicants to neurosurgery programs in the application years 2021–2022 as compared to 2019–2020.^15^ Use of social media accounts by residency programs also increased. In an evaluation of EM residency programs, an increase in social media use by 34% was documented following the onset of the COVID-19 pandemic in 2020.^5^ The most commonly used social media platforms by EM programs were X (75% of programs), Instagram (61% of programs) and Facebook (38%). Instagram use increased the most following the onset of COVID-19, with nearly half of all accounts created after March 2020, illustrating the dynamic nature of the use of these platforms in communicating with potential residency applicants.

Our descriptive analysis of resources used by applicants to learn about EM residency programs was in line with the trends from prior studies, yet some of our results were unexpected. Instagram use is rising and will likely surpass use of the EMRA Match website in the next interview season. The many other sites and online databases for students through our national organizations were rarely reported to be used.

Our “word-of-mouth” category included advising from mentors and conversations with peers. While it may seem surprising that this category was lower than the three resources above, the design of this study likely did not allow us to fully capture the impact of personal conversations. This free- text response measured frequency, without a way to measure the value of the information. Rotation experiences were also quite low. Some respondents may not have considered these to be “resources” and, therefore, did not consider typing “rotation” or “word of mouth” into our questionnaire.

## LIMITATIONS

The strength of our data lies in the sample size of 322 applicants over four years. However, a limitation is that the sampled cohort comes from a single residency program in the Northeast, established in 2019. Applicant responses may have differed if surveys were conducted at other programs across the country. Our method of data collection used free-text responses, which allowed students to type in the sources that first came to mind. Although this approach provided valuable insights, using a drop-down list or a menu of options might have been more efficient. Future research could include a multicenter study across various programs nationwide or a standardized question with a list of options to rank in order of preference, possibly integrated into commonly used scheduling platforms, such as Thalamus or the Electronic Residency Application Service.

## CONCLUSION

Our descriptive study of reported resources used by applicants to learn about residency programs showed that the program website remains the top resource and that Instagram and the EMRA Match website are also frequently used. Other websites and online databases, as well as program fairs at conferences, are rarely reported by applicants as providing important information. These findings might help program leaders to direct their efforts towards those resources that might have the greatest impact on recruitment.

## Supplementary Information
