# Supplementary material for: Descriptive Analysis of Resources Used to Learn About Residency Programs Since Transition to Virtual Interviews
Source: West J Emerg Med. 2025 May 19;26(3):569–72. doi: 10.5811/westjem.33574 (PMC12208053; doi:10.5811/westjem.33574)
Supplement: Supplementary file 1 [file wjem-26-569-s001.docx]

**Appendix [Appendix not called out in manuscript, please call out Appendix in body of manuscript.]**

**Custom Dictionary:**

Residency Website:

| Website | UVM | UVMMC | Larner |
| --- | --- | --- | --- |

EMRA:

| EMRA | Match |
| --- | --- |

Personal:

| PGY | Friend | Faculty | Resident | Alumni | Classmate |
| --- | --- | --- | --- | --- | --- |
| Personal | Conversation | Experience | Interview | Mentor | Advisor |
| Experience | Doctor | Applicants | Visit | Mouth | Student |
| Spoke | Chair | Peer | Director |  |  |

Rotation:

| Rotate | Rotation | Internship | TELL-EM |
| --- | --- | --- | --- |

Conference:

| Conference | SAEM | ACEP | Fair | Meeting |
| --- | --- | --- | --- | --- |

Stand-alone Words:

| Instagram | Twitter | Residency Explorer |
| --- | --- | --- |
| NRMP | Doximity | AAMC |
| Reddit | FREIDA |  |
